# Pain and Inflammation: Update on Emerging Phytotherapy, Zootherapy, and Nutritional Therapies

**DOI:** 10.1155/2016/2520371

**Published:** 2016-07-18

**Authors:** Bamidele V. Owoyele, Musa T. Yakubu, Roi Treister

**Affiliations:** ^1^Neuroscience and Inflammation Research Unit, Department of Physiology, University of Ilorin, Ilorin 240003, Nigeria; ^2^Phytomedicine, Toxicology and Reproductive Research Laboratory, Department of Biochemistry, University of Ilorin, Ilorin 240003, Nigeria; ^3^Department of Neurology, Massachusetts General Hospital and Harvard Medical School, Boston, MA 02114, USA

Pain is the most common health challenge that drives patients to consult physicians. Pain and inflammation are usually symptoms of many diseases. Inability to perceive sensory pain may lead to shortened life expectancy and yet excessive pain may result in low quality of life. Despite the arrays of therapies available for the treatment of pain, it is still not tamed and the currently available drugs have their drawbacks. The challenges to amelioration of pain and inflammation have become an impetus for research into alternative and complementary medicines for the treatment of these ailments. The roles of phytotherapy and nutritional therapies in the treatment of varied types of ailments cannot be overemphasized. Actually, most of the commonly available therapeutic drugs for pain, such as opioids and nonsteroidal anti-inflammatory drugs (NSAIDs), were derived from phytotherapy. Hence, giving attention to this issue is highly recommended as it provides a medium for knowledge acquisition for improvement of the treatment of the twin ailment-pain and inflammation.

The researches published in this issue are broad and they accomplished the aim for which the issue was set out. Many manuscripts were received but only those contributing significantly to the subject matter were accepted after thorough peer review. The new insights include topics on systemic models of inflammation contributed by Y. Liu et al., “Effects of Wutou Decoction on DNA Methylation and Histone Modifications in Rats with Collagen-Induced Arthritis,” and X. Wu et al., “Protective Effect of Tetrandrine on Sodium Taurocholate-Induced Severe Acute Pancreatitis.” Z. Shu et al. reported the anti-inflammatory effects of plant extract “Antibacterial and Anti-Inflammatory Activities of* Physalis alkekengi* var.* franchetii* and Its Main Constituents” as well as R. Boukhary et al., “Anti-Inflammatory and Antioxidant Activities of* Salvia fruticosa*: An HPLC Determination of Phenolic Contents.” In another study, I. J. M. Santos et al. reported “Topical Anti-Inflammatory Activity of Oil from* Tropidurus hispidus* (Spix, 1825).” A review was accepted for inclusion in the issue on effectiveness of acupuncture for treating sciatica by Z. Qin et al. A. Rauf et al. reported on a potential cyclooxygenase inhibitor named daturaolone while W. Kim et al. evaluated the anti-inflammatory potential of a new Ganghwaljetongyeum (N-GHJTY) on adjuvant-induced inflammatory arthritis in rats. This issue therefore is a rich resource for all interested in pain and inflammation research and treatment.

*Bamidele V. Owoyele* *Bamidele V. Owoyele* *Musa T. Yakubu* *Musa T. Yakubu* *Roi Treister* *Roi Treister*

